# Relative validity and reproducibility of a short semi-quantitative food frequency questionnaire for Chinese athletes

**DOI:** 10.1371/journal.pone.0317370

**Published:** 2025-01-09

**Authors:** Qian Xu, Yudan Chu, Huajun Tian, Guoqiang Ma, Jun Qiu, Qiuping Zhang

**Affiliations:** Key Laboratory of General Administration of Sport for Exercise Performance Evaluation, Shanghai Research Institute of Sports Science (Shanghai Anti-Doping Agency), Shanghai, China; Brigham and Women’s Hospital, UNITED STATES OF AMERICA

## Abstract

A convenient but efficient tool for evaluating dietary intakes in Chinese professional athletes has yet to be established. The aim of this study was to assess the validity of a short semi-quantitative food frequency questionnaire (FFQ) through comparison with 3-day weighed food records (3DWFRs) and corresponding serum biomarkers from a cohort of 102 professional athletes, while also evaluating its reproducibility. The relative validity was assessed using Spearman correlation coefficients, cross-quintiles classification, weighted kappa, and Bland–Altman analysis, while reproducibility was evaluated using the Spearman correlation coefficients and intraclass correlation coefficient (ICC) between two FFQs. The results showed the median (range) crude correlation coefficients (CCs) between the first FFQ and 3DWFRs for energy and nutrients, and the food groups were 0.331 (0.219 to 0.568) and 0.292 (-0.035 to 0.455), respectively. Omega-3 polyunsaturated fatty acid (EPA, DHA, and EPA+DHA) intake estimated from the FFQ significantly correlated with corresponding serum biomarkers, with CCs ranging from 0.389 to 0.520. Weighted kappa statistics, indicating acceptable consistency (> 0.2) for most items, varied from -0.076 to 0.581, except for vitamin C, vegetables, and oils. Misclassification of nutrients and food groups into extreme quartiles was rare, with a median misclassification rate of 2% (ranging from 1% to 12%) and 3% (ranging 0 to 10%), respectively. Bland–Altman analysis revealed good agreement between FFQ and 3DWFRs, with over 90% of data points falling within the limits of agreement (LOA) for all assessed nutrients and food groups. In the reproducibility analysis, the median (range) crude CC and ICC for energy and nutrients were 0.574 (0.423 to 0.643) and 0.668 (0.558 to 0.763), respectively, while for food groups, they were 0.681 (0.242 to 0.764) and 0.640 (0.371 to 0.787), respectively. In conclusion, the short FFQ demonstrated acceptable relative validity and reproducibility for most nutrients and food groups, suggesting its potential as a valuable tool for assessing dietary intake and nutrition status among young Chinese athletes.

## Introduction

A meticulously balanced and targeted nutritional regimen is essential for maintaining athletes’ health and optimal performance [1]. Given the diverse nutritional needs across various stages of training, routine dietary assessments are imperative to enable athletes to fine-tune their nutritional strategies accordingly [2]. However, conventional approaches to dietary assessment for athletes remain challenging, primarily due to the rigorous demands of training schedules and the logistical constraints imposed by off-site training sessions and competitive venues [3]. Therefore, it is crucial to find a convenient yet robust nutritional assessment tool capable of regular nutrient intake monitoring. Simultaneously, by examining the correlation between dietary patterns and athletic performance, we can identify effective nutritional intervention strategies for athlete cohorts.

Among the many dietary assessment methods available, food frequency questionnaires (FFQs) have been widely applied in nutritional epidemiology studies [4–6]. In addition to advantages such as rapid feedback, cost-effectiveness, and ease of administration, FFQs also excel in evaluating long-term habitual dietary intakes, rendering them invaluable for monitoring nutritional risks over time and exploring dietary patterns among various populations [7]. However, an FFQ must be validated for the target population prior to its application [8]. Considering the unique dietary habits among athletes across different countries, it is very necessary to develop country-specific FFQs and conduct rigorous methodological validation. This validation process should not only account for social and cultural contexts but also consider factors such as doping prevention measures and the use of nutritional supplements [9].

While the development and validation of FFQs for athlete populations in countries such as Japan, Brazil, and Croatia have been reported [10–13], an FFQ tailored for Chinese athletes has yet to be established, to the best of our knowledge. Given the substantial disparities in dietary habits and cultural backgrounds between these countries and China, the suitability of such reported FFQs for Chinese athletes is questionable. Moreover, existing reported FFQs for athletes often include an extensive array of items [10,11], potentially deterring athlete participation in research endeavors. Thus, this study aimed to design a shorter FFQ questionnaire. Its relative validity was assessed among Chinese professional athletes, utilizing weighed food records and biomarkers as reference methods. Additionally, a reproducibility analysis was conducted to evaluate the consistency of nutrient and food category intake measurements.

## Methods

### Participants and study design

**Fig 1:**
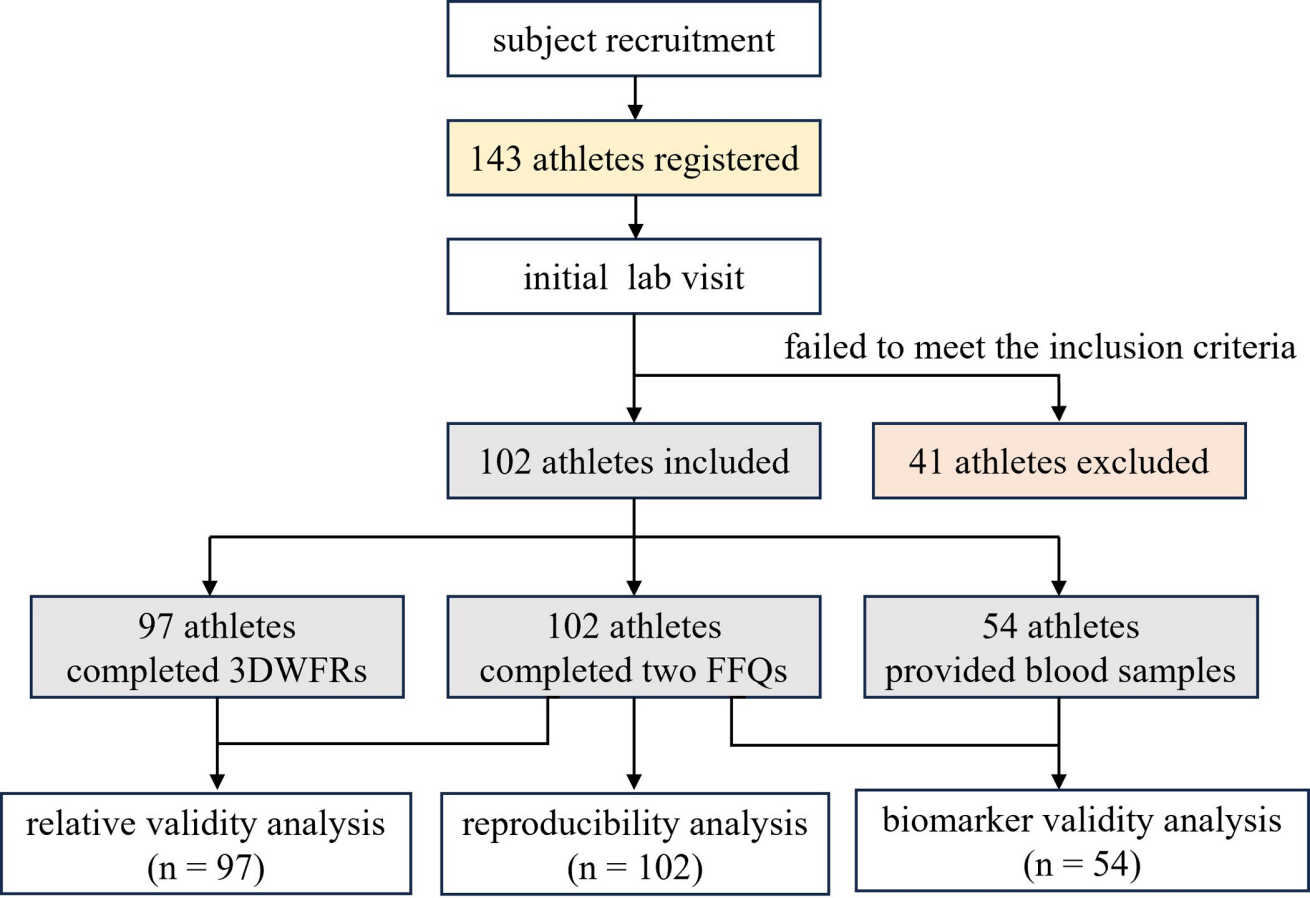
Flow chart of subject selection and determination. WFR, weighed food record; FFQ, food frequency questionnaire.

This cross-sectional study involved healthy professional athletes aged 14 to 30, conducted by the Shanghai Research Institute of Sports Science from 2022 to 2023. Participants were recruited via posters distributed among Shanghai’s professional sports teams from September 4 to November 20, 2022. Inclusion criteria required athletes to be at least second-level competitors, ranked within the top 24 for provincial and local sports bureau-organized championships, regularly dining in the training base’s designated restaurants, and free from known illnesses or recent injuries. After initial interest from 143 athletes, 41 were excluded following laboratory interviews due to irregular eating patterns, age restrictions, recent medication intake, or refusal to participate in a three-day weighing protocol. The final cohort consisted of 102 professional athletes, 49 males and 53 females, with a mean age of 20 years. Sports disciplines included cycling, track and field, handball, badminton, wushu, gymnastics, volleyball, swimming, boxing, shooting, and archery. Ethical approval was obtained from the Ethics Committee of the Shanghai Research Institute of Sports Science (LLSC20220021), and written informed consent was provided by all participants prior to the study’s commencement. The flow chart of subject selection and determination is illustrated in Fig 1.

**Fig 2:**
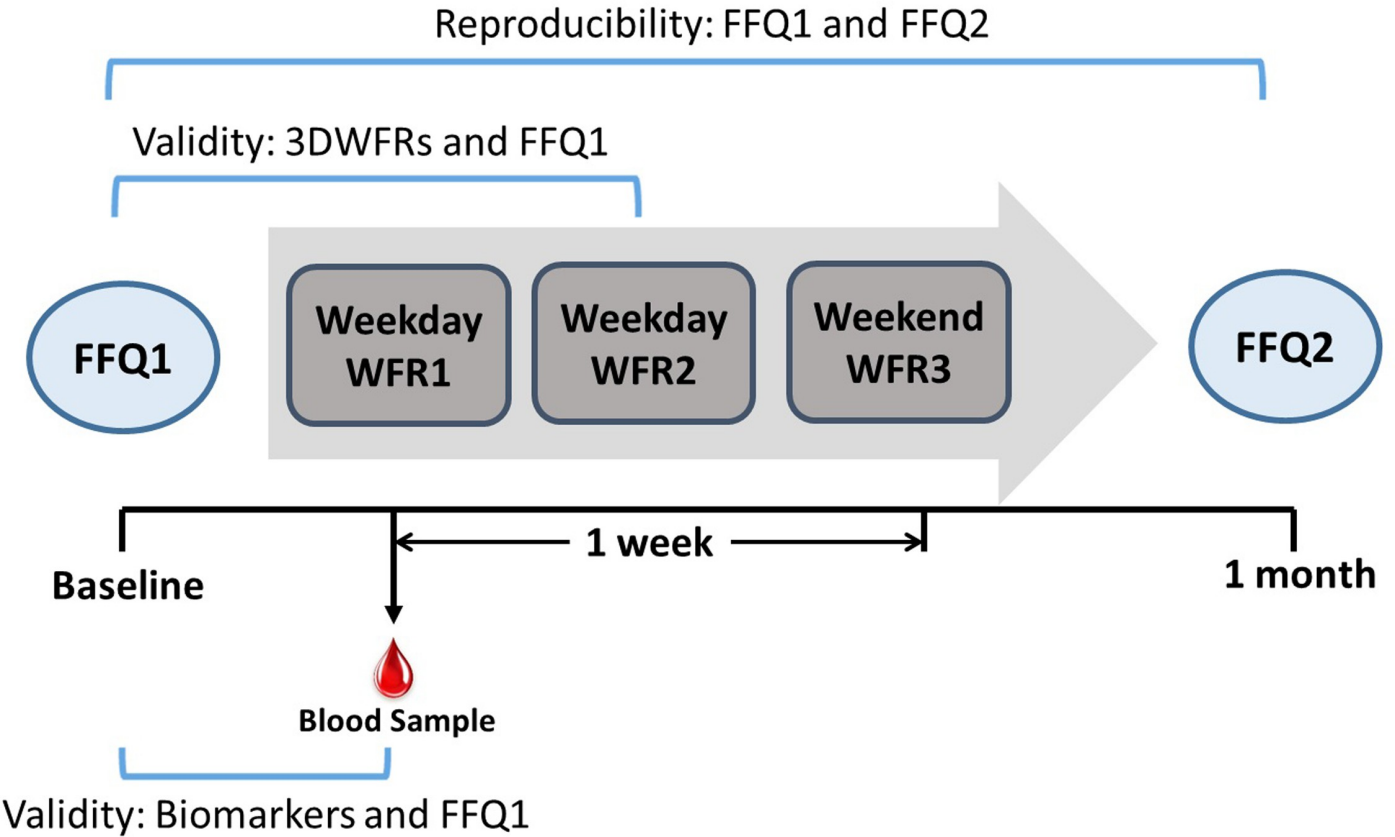
Study design. WFR, weighed food record; FFQ, food frequency questionnaire; WFR1 and WFR2: In training status; WFR3: In rest status.

The formal study was conducted during the winter training period from December 2022 to February 2023, a critical training phase for athletes. Evaluating dietary intake during this period provides insight into off-season nutrition status. Each participant completed the FFQ twice with an interval of one month in a self-reported fashion during the study period. Three nonconsecutive days (consisting of two training days (weekdays) and one rest day (weekend)) were selected in the second week of the interval month for three-day weighed food records (3DWFRs). On the morning of the 1^st^ weighing day, weighing equipment and instructions were distributed to the participants, and fasting venous blood samples were also obtained. The design of this study is shown in Fig 2. Of these 102 participants, 5 were excluded due to incomplete weighing data, and ultimately, 97 participants were included in the relative validity analysis of the FFQ versus the 3DWFRs, while all 102 participants were included in the reproducibility analysis of the two FFQs. Due to experimental funding constraints, only 54 blood samples that were randomly selected from the 102 samples were used for biomarker determination, and relative validity analysis of the FFQ versus blood biomarkers (n = 54) were conducted.

### Food frequency questionnaire

The short semi-quantitative FFQ questionnaire in this study was adapted from the 25-item FFQ questionnaire developed by Gao et al. for the middle-aged and elderly population in the Changfeng Study in Shanghai [14], reviewing their customary dietary intakes over the past year. In the present study, the recall duration of the FFQ was modified from 1 year to 1 month, mainly considering that athletes usually change their dietary strategies with different training periods, which makes short-term dietary assessment better meets practical needs. Besides, due to the specialty of athletes’ diets and the potential role of nutrients in athletes’ health and performance, four more items (legumes, instant foods, sports drinks and protein powder) and one open-ended question related to the use of nutritional supplements were added to this FFQ questionnaire. Once an answer of “yes” was provided for nutritional supplement use, the athletes were required to report in detail the brand and specification of the supplement, the dosage, the usage period, and the frequency of use. The nutrient content provided by the supplement was calculated from the above information. Finally, this FFQ questionnaire consisted of 29 items and one open-ended question. The 29 items were flour, rice, stuffed rice noodles, whole grains and their products, potatoes and their products, soybeans and their products, legumes, green vegetables, dark vegetables, light-colored vegetables, mushrooms and algae, fresh fruits, nuts, livestock meat, poultry, dairy products, eggs, freshwater fishes, seafood, desserts, instant foods, puffed foods, candied fruit, sweetened beverages, beer, yellow rice wine, liquor, sports drinks, and protein powder. The participants were asked how often they consumed each food and how many servings they consumed each time. Frequency included nine options: “never”, “less than 1 time per month”, “1–3 times per month”, “1–2 times per week”, “3–4 times per week”, “5–6 times per week”, “once per day”, “twice per day” and “3 or more times per day”, portion sizes were given in easy-to-assess units of measurement, such as “fist”, “palm”, “piece”, “handle”, and common vessel units such as “shovel”, “bag”, “cup”, “bottle”, and “spoon” to reduce participant burden. These units were eventually converted to intake values for each item based on experience and laboratory measurements. For the fixed food items in the FFQ, the daily food intake was the product of multiplying the frequency of daily food intake by the quantity of each food intake. The energy and nutrient content of each food item were based on the “Chinese Food Composition Table (6th Edition)” [15], calculated from the average values of energy and nutrients from 3–10 representative foods that constituted the item. These foods were identified through survey on the most frequently purchased ingredients in athletes’ cafeterias, combined with findings from our previous dietary surveys specifically conducted for athletes.

### Three-day weighed food records

Each participant was given a portable food scale and a number of disposable utensils for serving food, and the details of the operation were explained to the participants both verbally and in writing. Briefly, we asked the participants to serve and weigh each dish individually and then record the values of weighing data by taking photos with their cell phones and uploading these data to a designated folder together with the picture of each dish, in addition to filling in the name of the dish and the ingredients of the food mix. At the end of a meal, the participants were instructed to weigh and photograph the uneaten food in the same way. If there was no leftover food, there was no need to upload any more information. The weights of disposable tableware were obtained in advance so that the participants did not need to tare or repeat the weighing of empty bowls to minimize operational errors and reduce participant burden. On weekdays, the participants generally ate meals at designated cafeterias, and we had obtained the preparation ratios of all dishes in advance and loaded them into the Nutritional Calculator program; on weekends, the participants who might leave the training base to eat a meal were asked to carry food scales with them, weigh the food in the same way as on weekdays, upload photos of the food, and describe in detail the ingredients of the mixture and the approximate proportions of the food. Dietitians checked the folders at mealtimes and asked the participants to reshoot, re-upload, or re-fill if the photos were unclear or there was missing information to ensure that the results were accurate and valid. Finally, the data from the 3DWFRs were calibrated by four trained dietitians and input into the Nutrition Calculator program (Beijing Bowen Information Technology Co., Ltd.), which automatically calculates and summarizes daily energy and nutrient intake based on the latest Chinese Food Composition Table (6th edition).

### Blood biomarkers

Venous blood samples were taken from the fasting subjects and placed in vacuum tubes without anticoagulant. Serum was separated via centrifugation at 3000× g for 15 min, and multiple aliquots of each sample were stored at -80°C for subsequent analysis of serum fatty acids. Serum total fatty acids were extracted using the fatty acid methylation kit^®^ produced by Sigma–Aldrich in accordance with their instructions. The lipid assay was conducted using a GC–MS/MS system (Agilent 7010B, USA) to quantitate serum fatty acids via precursor ion scanning for the m/z values corresponding to the respective molecular weights. Calibration curves with standards showed a very good correlation between the concentration and response. All the calibration curves were reproducible. The concentration of each individual fatty acid was expressed as a percentage of the total area under the peaks.

### Statistical analysis

The average intakes of energy, nutrients, and food groups from the three weighed food records were used as a reference for dietary intake. Data are expressed as medians and interquartile ranges because most of the variables were not normally distributed. The difference in mean values between the 3DWFRs and FFQ was calculated as follows: (FFQ-3DWFR)/3DWFR*100%; additionally, the intake values between the two methods were compared using the Wilcoxon rank sum test. The relative validity of the FFQ in question for assessing the intake of energy, nutrients, and food groups was investigated using crude Spearman’s correlation coefficients, energy-adjusted correlation coefficients, and de-attenuated correlation coefficients. The residual method proposed by Willett et al. was used to calculate the energy-adjusted variables [16], and the de-attenuated correlation coefficients were calculated in accordance with Rosner et al.’s method [17]. The relationship between the intake of fatty acids estimated from the FFQ and serum biomarkers was investigated using the Spearman’s correlation analyses. In this study, items with coefficients exceeding 0.30 were considered to demonstrate acceptable validity, where values of 0.30–0.39 indicated fair validity, 0.40–0.59 indicated moderate validity, and ≥ 0.60 indicated high validity [11]. The agreement between the FFQ and 3DWFRs was evaluated by classifying the participants according to their distributions into quintiles of energy, nutrients, and food groups for each method. The percentages of athletes who were classified into the same, adjacent, and extreme quintiles were estimated. Kappa statistics were also calculated, and the weighted kappa values exceeding 0.2 were considered acceptable [18,19]. The level of agreement for energy and macronutrient intake estimates between the two methods was also evaluated using the Bland–Altman method by building scatterplots with the mean intake and the absolute difference in intake of the two methods. Plots showed the lines for the mean difference of intakes and the limits of agreement (LOA), defined as mean difference ± 1.96 SD. In the reproducibility test, Spearman’s correlation coefficients, energy-adjusted correlation coefficients, and intraclass correlation coefficients (based on a meanrating (k = 2), absolute-agreement, 2-way mixed-effects model) were calculated between the first and second FFQ. The following classification was used: poor reproducibility: < 0.50, moderate reproducibility: 0.75–0.90 and excellent reproducibility: > 0.9 [20]. P values less than 0.05 were considered statistically significant. All statistical analyses were conducted using IBM SPSS Statistics ver. 27.

## Results

The characteristics of the study population are shown in Table 1. The characteristics separated by gender can be found in S1 Table. Among the study participants, 52% were female athletes, with an average age of 20 years and an average BMI of 22.5 kg/m^2^. The athletes represented 11 different sports disciplines, with 47.1% being national-level athletes, 28.4% being first-level athletes, and 24.5% being second-level athletes. The average amount of training experience was 8.5 years, and the average weekly training time was 28.4 hours.

**Table 1:** Characteristics of the 102 study participants.

Characteristics	Results
Sex (n, %)	
Male	49 (48.0)
Female	53 (52.0)
Age, y (mean±SD)	20.0 ± 4.0
Height, cm (mean±SD)	173.9 ± 8.6
Weight, kg (mean±SD)	68.4 ± 15.2
Body Mass Index, kg/m^2^ (mean±SD)	22.5 ± 4.0
Sports Disciplines (n, %)	
Cycling Track and Field	17 (16.7) 14 (13.7)
Handball Badminton Wushu Gymnastics Volleyball Swimming Boxing Shooting Archery	8 (7.8) 6 (5.9) 13 (12.8) 6 (5.9) 3 (2.9) 5 (4.9) 5 (4.9) 8 (7.8)) 17 (16.7)
Athlete Class (n, %)	
National^a^	48 (47.1)
1^st^ Level^b^	29 (28.4)
2^nd^ Level^c^	25 (24.5)
Training years, y (mean±SD)	8.5 ± 3.6
Weekly Training Time, h (mean±SD)	28.4 ± 9.0

^a^ Athletes competing at the national level who are eligible to compete in comprehensive competitions and championships held by national sports bureaus and satisfy the criteria.

^b^ The top 12 competitors who are eligible to compete in comprehensive competitions and championships organized by provincial and local sports bureaus and have been designated as first level athletes.

^c^ The top 24 competitors who are eligible to compete in comprehensive competitions and championships organized by provincial and local sports bureaus and have been designated as second level athletes.

Table 2 presents the relative validity of the energy, nutrients, and food groups between the 3DWFRs and 1^st^ FFQ. For energy and nutrients, FFQ overestimated the intake of most nutrients, except for fat, vitamin A, vitamin E, sodium, and iron. Based on the percentage differences, the intake differences between the two methods for energy, carbohydrates, dietary fiber, cholesterol, vitamin A, vitamin E, calcium, potassium, magnesium, iron, and manganese were relatively small (percentage difference <10%). However, significant differences (p<0.05) were observed in the intake of vitamin B1, vitamin B2, niacin, vitamin C, Vitamin D, sodium, magnesium, zinc, selenium, and copper, with percentage difference from 23.13% for vitamin B2 to 905.81% for Vitamin D. For food groups, the FFQ overestimated the intake of fruits, poultry and meat, milk and dairy products, and legumes and nuts while underestimating the intake of cereals and potatoes, vegetables, fish and shellfish, eggs, and oils. Except for fish and shellfish, significant differences in the intake of food groups were observed between the two methods, with percentage difference ranging from 10.91% to 146.19%.

**Table 2:** Comparison of the daily nutrient and food group intakes from 3DWFR and FFQ1 and the corresponding validity correlation coefficients (n = 97).

	3DWFR^a^		FFQ^b^		%Difference^c^	*p* ^d^	Correlation Coefficient		
	Median	(Interquartile range)	Median	(Interquartile range)			crude^e^	Energy-adjusted^f^	Deattenuated^g^ (95% CI)
**Energy and nutrients** Energy (kcal)	2019.00	(1595.00, 2603.00)	2169.93	(1453.86, 2654.21)	3.13 (-31.83, 36.54)	0.726	0.384**	0.384**	0.390 (0.061, 0.450)
Protein (g)	89.80	(70.10, 122.65)	114.75	(66.08, 157.90)	11.43 (-28.75, 79.37)	0.007	0.330**	0.262**	0.217 (-0.320, 0.106)
Fat (g)	86.70	(69.30, 118.00)	76.36	(48.17, 105.52)	-16.26 (-46.19, 31.29)	0.072	0.350**	-0.106	-0.028 (-0.045, 0.379)
Carbohydrate (g)	218.63	(172.20, 267.00)	229.91	(165.99, 315.34)	5.78 (-28.15, 52.03)	0.178	0.318**	0.164	0.111 (0.061, 0.473)
Dietary fiber (g)	8.30	(6.10, 11.44)	9.25	(6.25, 12.37)	1.33 (-30.73, 75.74)	0.336	0.256*	0.259*	0.429 (0.149, 0.543)
Cholesterol (mg)	566.00	(375.25, 896.25)	586.25	(366.71, 856.28)	4.81 (-28.53, 42.05)	0.644	0.568**	0.334*	0.437 (-0.042, 0.387)
Vitamin A (μg)	456.00	(265.00, 649.00)	366.86	(259.60, 570.03)	-7.88 (-40.82, 23.76)	0.053	0.328**	0.168	0.161 (-0.112, 0.330)
Vitamin B1 (mg)	1.08	(0.71, 1.35)	1.47	(0.92, 2.02)	36.5 (1.86, 111.44)	< 0.001	0.333**	0.104	0.055 (-0.007, 0.435)
Vitamin B2 (mg)	1.11	(0.83, 1.59)	1.40	(0.92, 2.03)	23.13 (-17.98, 83.59)	< 0.001	0.419**	0.200*	0.234 (0.054, 0.468)
Niacin (mg)	18.58	(13.75, 25.29)	25.51	(16.33, 36.50)	36.49 (-13.14, 123.63)	< 0.001	0.309**	0.252*	0.184 (0.024, 0.443)
Vitamin C (mg)	69.20	(41.10, 103.50)	114.52	(72.90, 164.79)	63.6 (-6.67, 169.17)	< 0.001	0.219*	0.226*	0.354 (-0.114, 0.336)
Vitamin E (mg)	19.40	(14.50, 25.81)	18.70	(13.43, 26.84)	-1.06 (-31.71, 34.87)	0.683	0.253*	0.104	0.151 (-0.141, 0.311)
Vitamin D (μg)	2.85	(1.30, 4.80)	27.25	(13.18, 46.13)	905.81 (287.13, 2175.37)	< 0.001	0.315**	0.080	0.066 (0.066, 0.515)
Calcium (mg)	566.00	(380.00, 833.00)	605.57	(420.75, 911.72)	7.76 (-22.63, 90.02)	0.092	0.321**	0.258*	0.274 (0.198, 0.584)
Phosphorus (mg)	1086.30	(831.00, 1421.75)	1174.69	(779.11, 1526.62)	13.84 (-29.61, 48.11)	0.054	0.404**	0.373**	0.305 (0.043, 0.454)
Potassium (mg)	1963.30	(1401.00, 2506.00)	1959.54	(1320.56, 2587.86)	9.31 (-33.45, 50.49)	0.318	0.332**	0.244*	0.356 (-0.126, 0.309)
Sodium (mg)	3012.40	(2220.34, 3876.10)	2037.23	(1327.22, 2576.75)	-37.51 (-56.93, -7.45)	< 0.001	0.376**	0.089	0.116 (0.061, 0.468)
Magnesium (mg)	283.00	(200.00, 374.00)	321.51	(221.24, 399.14)	8.15 (-21.55, 61.35)	0.049	0.360**	0.259*	0.355 (0.092, 0.494)
Iron (mg)	19.00	(13.70, 26.60)	18.07	(12.44, 24.90)	-6.42 (-34.75, 43.34)	0.745	0.330**	0.287**	0.301 (-0.013, 0.415)
Zink (mg)	12.26	(8.53, 16.85)	15.55	(10.49, 21.35)	27.9 (-21.49, 104.63)	0.001	0.331**	0.194	0.223 (0.052, 0.468)
Selenium (μg)	47.40	(35.35, 71.05)	67.04	(41.31, 88.27)	31.56 (-24.5, 102.63)	0.001	0.416**	0.251*	0.218 (-0.023, 0.398)
Copper (mg)	1.18	(0.84, 1.61)	1.79	(1.23, 2.27)	41.03 (-7.27, 126.27)	< 0.001	0.339**	0.184	0.301 (-0.106, 0.365)
Manganese (mg)	3.12	(2.19, 4.06)	3.37	(2.50, 4.62)	3.67 (-22.09, 64.57)	0.07	0.269*	0.116	0.290 (1.006, 1.454)
**Food groups** Cereals and potatoes (g)	375.40	(274.10, 523.00)	332.74	(197.89, 437.48)	-14.28 (-47.82, 24.72)	0.002	0.355**	0.224*	0.266 (0.022, 0.480)
Vegetables (g)	206.39	(140.00, 260.20)	125.00	(75.00, 217.25)	-40.32 (-61.75, 5.26)	< 0.001	0.193	0.197	0.457 (-0.010, 0.420)
Fruits (g)	44.20	(0.00, 142.62)	228.00	(114.00, 360.24)	146.19 (13.1, 491.28)	< 0.001	0.292**	0.373**	0.409 (0.192, 0.569)
Poultry and meat (g)	220.50	(162.10, 348.30)	309.90	(159.90, 543.25)	37.54 (-30.06, 147.74)	< 0.001	0.375**	0.198	0.327 (-0.009, 0.413)
Fish and shellfish (g)	58.90	(11.80, 111.20)	42.90	(13.65, 96.53)	-53.27 (-77.01, 28.99)	0.055	0.287**	0.151	0.208 (-0.059, 0.366)
Eggs (g)	88.10	(47.10, 136.30)	33.00	(16.50, 66.00)	-55.27 (-79.82, -14.46)	< 0.001	0.455**	0.212*	0.320 (0.007, 0.424)
Milk and dairy products (g)	161.70	(15.20, 278.00)	158.00	(88.00, 300.00)	10.91 (-40.01, 125.91)	0.029	0.377**	0.314**	0.388 (0.125, 0.527)
Legumes and nuts (g)	8.70	(3.30, 17.10)	11.00	(3.50, 33.00)	65.67 (-64.54, 303.96)	0.004	0.187	0.118	0.235 (-0.096, 0.341)
Oils (g)	21.00	(13.70, 29.20)	10.57	(5.35, 21.00)	-43.15 (-77.26, 12.1)	< 0.001	-0.035	-0.218*	-0.196 (-0.430, -0.014)

* Significant values (p < 0.05)

** Significant values (p < 0.01).

^a^ Intakes based on 3-day weighed food records.

^b^ Intakes based on the first FFQ.

^c^ Percentage difference = [(FFQ-3DWFR)/3DWFR]*100.

^d^ Wilcoxon rank sum test for comparing energy and nutrient intake from 3DWFR and the FFQ.

^e^ Spearman’s correlation coefficients.

^f^ Energy-adjusted correlation coefficients that were calculated using a residual method.

^g^ Deattenuated CCx = observed CCx * SQRT (1 + λx/n), where λx is the ratio of within- to between-individual variance for nutrient x, and n is number of weighed food records; observed CCs were based on energy-adjusted values.

The Spearman’s crude correlation coefficients (CCs) for energy and most nutrients were in the range of 0.3–0.5 (83% of nutrients had crude CCs exceeding 0.3). The median crude CC was 0.331, ranging from 0.219 for vitamin C to 0.568 for cholesterol. After energy adjustment, the CCs of most nutrients decreased, except for dietary fiber and vitamin C. The median energy-adjusted CC and deattenuated CC for energy and nutrients were 0.226 and 0.234, respectively. For food groups, Spearman’s crude CCs of cereals and potatoes, poultry and meat, eggs, and milk and dairy products exceeded 0.3, with eggs demonstrating the highest CC of 0.455. Vegetables as well as legumes and nuts displayed relatively weaker crude CCs of 0.193 and 0.187, respectively, while oils exhibited the lowest crude CC of -0.035. After energy adjustment, the CCs of most food groups decreased, except for fruits. The median energy-adjusted CC and deattenuated CC for food groups were 0.198 and 0.320, respectively.

Table 3 shows the dietary intakes and serum levels of fatty acids from a subsample of participants (n = 54). The CCs between the dietary intakes of EPA, DHA, and EPA+DHA estimated from the 1st FFQ and corresponding serum levels were 0.389 (95% CI: 0.007, 0.672), 0.520 (95% CI: 0.171, 0.753), and 0.463 (95% CI: 0.097, 0.718), respectively.

**Table 3:** Correlation coefficients of fatty acids between dietary intake estimated from FFQ1 and serum biomarkers (n = 54).

Fatty acids	Serum Biomarkers (% of total fatty acids)	FFQ (g/d)	Spearman’s Correlation Coefficient (95% CI)
SFA	30.70 (29.67, 31.90)	16.25 (11.23, 30.13)	-0.057 (-0.431, 0.333)
MUFA	16.04 (15.25, 17.13)	21.55 (12.63, 32.28)	0.045 (-0.334, 0.411)
PUFA	53.29 (52.15, 54.99)	7.85 (6.18, 11.63)	-0.137 (-0.485, 0.249)
EPA	0.62 (0.41, 1.04)	0.0474 (0.0194, 0.1147)	0.389* (0.007, 0.672)
DHA	3.05 (2.71, 3.48)	0.0789 (0.0287, 0.1581)	0.52** (0.171, 0.753)
EPA+DHA	3.89 (3.11, 4.59)	0.1321 (0.0437, 0.2732)	0.463* (0.097,0.718)

* Significant values (p < 0.05)

** Significant values (p < 0.01).

SFA: Saturated fatty acid; MUFA: Monounsaturated fatty acid; PUFA: Polyunsaturated fatty acid; EPA: Eicosapentaenoic acid; DHA: Docosahexaenoic acid.

The results regarding individual ranking consistency between 3DWFDs and the first FFQ based on the distribution of energy, nutrients, and food groups across quintiles are shown in Table 4. For energy and nutrients, the median percentage of participants correctly classified into the same or adjacent quintiles was 61%, and extreme misclassification corresponded to 2%. Except for vitamin E (12%), the misclassification rates for energy and most nutrients were less than 10%. High consistency was observed for energy, fat, carbohydrates, cholesterol, phosphorus, potassium, sodium, magnesium, zinc, selenium, and copper (with extreme quintile proportions < 3%). For food groups, the median percentage of participants correctly classified into the same or adjacent quintiles was 61%, while the extreme misclassification percentage was 3%. High consistency was observed for fruits, poultry and meat, and eggs, while oils showed the worst consistency (10%). The weighted kappa statistics indicated acceptable consistency (> 0.2) for energy and most nutrients and food groups, except for vitamin C, vegetables, and oils.

**Table 4:** Agreement of cross-quintile classification and weighted kappa between 3DWFRs and FFQ1 (n = 97).

	Cross-Classification (%)			Weighted Kappa (95% CI)
	Same Quartile	Same or an Adjacent Quartile	Extreme Quartile	
**Energy and nutrients** Energy	28	63	1	0.343** (0.168, 0.519)
Protein	21	63	3	0.303** (0.122, 0.484)
Fat	28	62	2	0.343** (0.172, 0.515)
Carbohydrate	36	57	1	0.268** (0.084, 0.451)
Dietary fiber	27	58	3	0.264** (0.084, 0.444)
Cholesterol	43	72	1	0.581** (0.438, 0.723)
Vitamin A	36	64	4	0.293** (0.103, 0.483)
Vitamin B1	30	70	5	0.333** (0.145, 0.522)
Vitamin B2	27	68	3	0.361** (0.184, 0.538)
Niacin	34	56	1	0.268** (0.088, 0.447)
Vitamin C	22	60	5	0.197 (0.004, 0.39)
Vitamin E	38	61	12	0.321** (0.119, 0.523)
Vitamin D	21	64	3	0.336** (0.159, 0.513)
Calcium	25	59	4	0.253* (0.066, 0.439)
Phosphorus	31	55	1	0.345** (0.173, 0.517)
Potassium	22	57	1	0.283** (0.113, 0.453)
Sodium	27	60	2	0.338** (0.165, 0.512)
Magnesium	28	61	1	0.314** (0.138, 0.49)
Iron	30	59	3	0.308** (0.128, 0.488)
Zink	29	62	1	0.338** (0.167, 0.51)
Selenium	32	53	1	0.384** (0.215, 0.552)
Copper	29	61	1	0.349** (0.173, 0.525)
Manganese	28	64	4	0.241* (0.034, 0.448)
**Food groups** Cereals and potatoes	33	57	3	0.338** (0.16, 0.517)
Vegetables	26	59	5	0.193 (0.006, 0.381)
Fruits	28	60	0	0.286** (0.116, 0.456)
Poultry and meat	31	59	2	0.328** (0.155, 0.502)
Fish and shellfish	24	58	3	0.272** (0.096, 0.449)
Eggs	31	67	1	0.456** (0.299, 0.612)
Milk and dairy products	36	69	5	0.358** (0.165, 0.552)
Legumes and nuts	28	61	4	0.228* (0.037, 0.418)
Oils	18	48	10	-0.076 (-0.275, 0.122)

* Significant values (p < 0.05)

** Significant values (p < 0.01).

**Fig 3:**
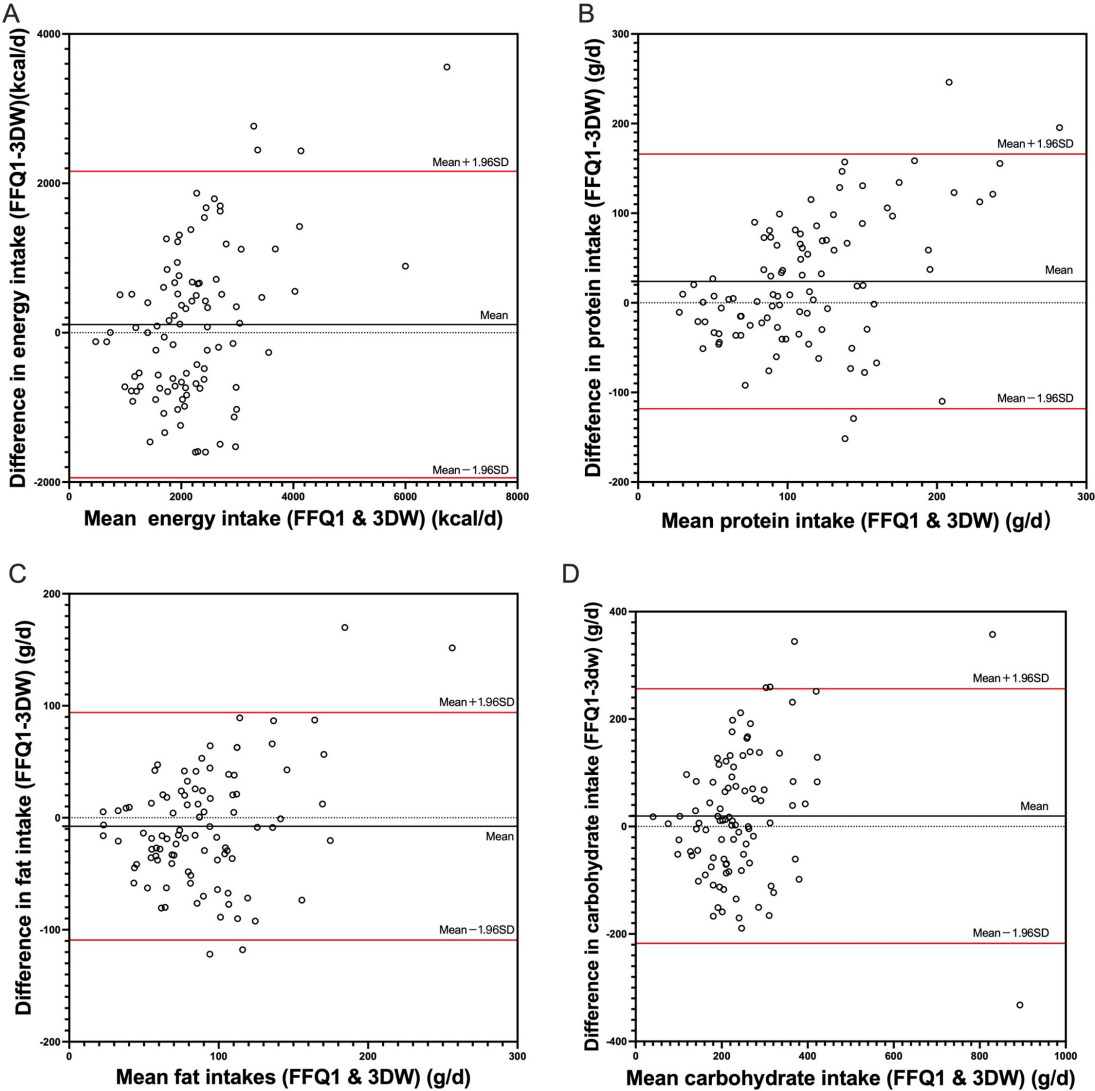
Bland–Altman plots showing the agreement between 3DWFRs and FFQ1 for (A) energy, (B) protein, (C) fat, and (D) carbohydrate (n = 97).

Bland–Altman analysis was used to assess the level of agreement by plotting the relationship of the mean and difference values for daily intakes between 3DWFR and the first FFQ. Fig 3 depicts the Bland–Altman plots for energy and macronutrients. In each plot, the solid black line represents the mean difference in intake between the FFQ and 3DWFRs, while the two solid red lines represent the limits of agreement (LOA, mean difference ± 1.96SD). For energy and macronutrients, most data points fall within the LOA, and the majority of data points are close to the mean line, indicating an acceptable level of consistency between the two methods. The Bland–Altman analysis data for energy, nutrients, and food groups are shown in S2 Table. The percentage of data points within the LOA is above 90% for all the nutrients and food groups.

Table 5 shows the reproducibility results for energy, nutrients, and food group intakes assessed via two FFQs. For energy and nutrients, the second FFQ underestimated the intake of most nutrients, except for cholesterol, vitamin A, and calcium. The median crude CC was 0.574, ranging from 0.423 for vitamin C to 0.643 for vitamins B1 and B2. After energy adjustment, the direction of change in the CC values varied across different nutrients, with a median energy-adjusted CC of 0.530. The median intraclass correlation coefficient (ICC) was 0.668, ranging from 0.558 (95% CI: 0.379, 0.697) for vitamin A to 0.763 (95% CI: 0.645, 0.845) for vitamin B1. For food groups, the second FFQ overestimated the intake of cereals and potatoes as well as fish and shellfish while underestimating the intake of vegetables, fruits, poultry and meat, and oils. The median crude CC, energy-adjusted CC, and ICC for food groups were 0.681, 0.632, and 0.640, respectively. Legumes and nuts consistently exhibited the lowest CCs (0.436, 0.242, and 0.371, respectively) across all analyses.

**Table 5:** Daily nutrient and food group intakes from FFQ1 and FFQ2 and reproducibility correlation coefficients (n = 102).

	FFQ1^a^		FFQ2^b^		Correlation Coefficient		
	Median	(Interquartile range)	Median	(Interquartile range)	crude^c^	Energy-adjusted^d^ (95% CI)	ICC^e^ (95% CI)
**Energy and nutrients** Energy	1916.00	(1131.16, 2769.18)	1751.95	(1280.42, 2566.04)	0.596***	0.596*** (0.418, 0.730)	0.697*** (0.558, 0.798)
Protein	101.06	(53.34, 153.45)	94.39	(58.43,145.35)	0.626***	0.684*** (0.534, 0.792)	0.687*** (0.544, 0.791)
Fat	66.70	(39.46, 95.29)	65.32	(41.90, 96.92)	0.594***	0.576*** (0.393, 0.715)	0.692*** (0.552, 0.795)
Carbohydrate	203.28	(144.08, 293.33)	202.99	(143.85, 306.99)	0.551***	0.644*** (0.481, 0.764)	0.680*** (0.535, 0.787)
Dietary fiber	8.52	(5.44, 11.75)	7.36	(5.60, 11.34)	0.520***	0.507*** (0.308, 0.664)	0.604*** (0.436, 0.731)
Cholesterol	492.18	(319.68, 845.03)	506.18	(294.50, 766.70)	0.534***	0.456*** (0.246, 0.625)	0.597*** (0.428, 0.726)
Vitamin A	350.66	(234.97, 572.41)	365.94	(212.75, 501.09)	0.541***	0.445*** (0.233, 0.617)	0.558*** (0.379, 0.697)
Vitamin B1	1.43	(0.83, 2.09)	1.24	(0.86, 1.73)	0.643***	0.444*** (0.232, 0.616)	0.763*** (0.645, 0.845)
Vitamin B2	1.25	(0.80, 1.88)	1.18	(0.83, 1.65)	0.643***	0.530*** (0.335, 0.681)	0.700*** (0.557,0.802)
Niacin	22.96	(13.50, 35.98)	19.90	(12.54, 34.98)	0.579***	0.586*** (0.405, 0.722)	0.720*** (0.584, 0.816)
Vitamin C	103.70	(73.19, 161.21)	88.53	(61.18, 137.03)	0.423***	0.380** (0.157, 0.565)	0.637*** (0.465, 0.760)
Vitamin E	18.35	(10.91, 24.75)	17.02	(10.45, 26.99)	0.550***	0.465*** (0.256, 0.632)	0.649*** (0.493, 0.764)
Vitamin D	20.47	(10.82, 40.21)	19.85	(11.06, 34.29)	0.564***	0.475*** (0.269, 0.640)	0.735*** (0.606, 0.826)
Calcium	551.36	(363.73, 827.47)	591.12	(360.14, 844.67)	0.519***	0.491*** (0.288, 0.652)	0.568*** (0.390, 0.705)
Phosphorus	1050.66	(639.81, 1497.46)	1013.39	(676.14, 1450.53)	0.591***	0.669*** (0.514,0.782)	0.651*** (0.497, 0.765)
Potassium	1854.62	(1179.16, 2522.71)	1742.83	(1169.06, 2430.34)	0.594***	0.572*** (0.388, 0.712)	0.668*** (0.518, 0.779)
Sodium	1965.06	(1061.8, 2447.76)	1758.68	(1150.07, 2290.76)	0.574***	0.435*** (0.222, 0.609)	0.751*** (0.630, 0.837)
Magnesium	297.31	(188.67, 394.83)	262.30	(180.45, 394.00)	0.606***	0.608*** (0.434, 0.738)	0.680*** (0.536, 0.786)
Iron	17.49	(10.40, 23.67)	15.78	(10.40, 23.57)	0.578***	0.598*** (0.421,0.731)	0.664*** (0.515, 0.775)
Zink	13.29	(8.11, 20.05)	12.64	(7.70, 19.57)	0.571***	0.400*** (0.180, 0.582)	0.621*** (0.458, 0.744)
Selenium	55.01	(34.09, 89.53)	53.59	(35.18, 79.92)	0.555***	0.526*** (0.330, 0.678)	0.602*** (0.434, 0.730)
Copper	1.71	(1.04, 2.30)	1.54	(1.03, 2.34)	0.588***	0.622*** (0.452, 0.749)	0.669*** (0.516, 0.779)
Manganese	3.01	(2.19, 4.28)	2.96	(2.14, 4.60)	0.525***	0.579*** (0.396, 0.717)	0.613*** (0.446, 0.739)
**Food groups** Cereals and potatoes	281.32	(160.44, 421.10)	298.05	(176.22, 433.50)	0.681***	0.641*** (0.396, 0.801)	0.640*** (0.407, 0.795)
Vegetables	115.50	(57.88, 184.94)	88.63	(52.50, 178.13)	0.654***	0.544*** (0.263, 0.740)	0.610*** (0.364, 0.776)
Fruits	228.00	(92.63, 342.00)	180.12	(114.00, 281.30)	0.712***	0.764*** (0.581, 0.873)	0.774*** (0.606, 0.876)
Poultry and meat	255.63	(137.58, 434.52)	210.36	(107.15, 457.68)	0.606***	0.401* (0.083, 0.644)	0.637*** (0.400, 0.794)
Fish and shellfish	35.10	(14.14, 85.07)	40.95	(13.65, 74.83)	0.752***	0.735*** (0.536, 0.857)	0.752*** (0.574, 0.862)
Eggs	26.07	(16.50, 50.00)	26.07	(12.50, 72.75)	0.716***	0.632*** (0.383, 0.795)	0.667*** (0.444, 0.812)
Milk and dairy products	200.00	(79.00, 300.00)	200.00	(84.25, 300.00)	0.767***	0.711*** (0.499, 0.843)	0.787*** (0.628, 0.883)
Legumes and nuts	11.00	(3.50, 44.00)	11.00	(3.50, 50.00)	0.436**	0.242 (-0.094, 0.529)	0.371* (0.059, 0.616)
Oils	9.47	(3.33, 21.48)	9.18	(5.08, 16.88)	0.611***	0.570*** (0.297, 0.756)	0.622*** (0.379, 0.784)

* Significant values (p < 0.05)

** Significant values (p < 0.01)

*** Significant values (p < 0.001).

^a^ Intakes based on the first FFQ.

^b^ Intakes based on the second FFQ.

^c^ Spearman’s correlation coefficients.

^d^ Energy-adjusted correlation coefficients that were calculated using a residual method.

^e^ Intraclass correlation coefficients.

## Discussion

A short FFQ comprising only 29 items and one open-ended question was created in this study to maximize athlete cooperation and compliance that can ultimately enhance the practicality and reliability of FFQs. The FFQ demonstrated acceptable validity and reproducibility across most nutrients and food groups, making it an effective tool for assessing the habitual nutrient intake of young athletes during specific training periods.

In this study, the weighed food records (WFRs) method was employed as the primary reference method for assessing the relative validity of the FFQ. Despite the cumbersome nature of weighing food, unlike the conventional 24-hour dietary recall method (24HDR), WFRs do not rely on memory accuracy, providing data closest to actual intakes, and can avoid the generation of homogeneity errors in FFQs [8,21,22]. Consequently, it offers a more robust approach to minimizing the potential to overestimate relative validity, thereby yielding reasonable validity results. Additionally, the dietary intake of fatty acids by athletes, especially omega-3 polyunsaturated fatty acids, was of particular interest to us because these acids have been reported to correlate with athletes’ inflammation levels and performance [23,24]. As the nutrition analysis software employed for analyzing the 3DWFRs data was unable to automatically aggregate the daily intake of fatty acids, blood biomarker measurement was introduced as an alternative reference method to evaluate the relative validity of fatty acid intakes. While assessing nutrient intake via blood biomarkers may be influenced by individual metabolic variations, it can effectively mitigate the inherent limitations of conventional dietary intake assessment methods, such as recall bias or subjectivity [25–27]. To the best of our knowledge, this is the first study to assess the validity of a FFQ for professional athletes simultaneously using both WFRs and biomarkers as reference methods.

A previous study that examined the validity of an FFQ including 138 foods, 20 beverages, and 14 flavorings in relation to Japanese college athletes showed that the median crude CCs for energy and nutrients in male and female athletes were 0.29 and 0.36, respectively [11]. Another similar study conducted on Japanese athletes demonstrated a median crude CC of 0.407 for nutrients (ranging from 0.222 for dietary fiber to 0.550 for carbohydrates), employing an FFQ comprising 62 food items and four supplement questions [13]. In comparison to our study, these investigations utilized longer FFQs. In addition, a meta-analysis assessing the relative validity of FFQs among healthy adults indicated that longer FFQs covering a broader spectrum of food items tended to exhibit stronger correlations with reference methods compared to shorter FFQs [8]. However, the final analysis results of this study indicated that our concise FFQ provided CCs for energy and most nutrients that were comparable to or slightly lower than those observed with longer FFQs. In this investigation, 83% of nutrients exhibited crude CCs exceeding 0.3, with a median CC of 0.331. Among them, acceptable to moderate levels of validity (CCs = 0.315–0.568) were achieved for the energy and macronutrients that were directly related to athletes’ health and performance, such as protein, carbohydrates, fat, and cholesterol, as well as for the micronutrients of interest, including vitamin D, calcium, and iron. However, the CCs for most nutrients decreased after energy adjustment, which may be attributed to the large differences in energy intake among individuals and systematic errors for energy intake from either or both the FFQ and 3DWFR.

Additionally, the assessment of omega-3 polyunsaturated fatty acids (EPA, DHA, and EPA+DHA) through the FFQ demonstrated significant correlations with corresponding serum biomarkers. Previous research employing Sublette et al.’s 21-item omega-3 FFQ [28] reported CCs of 0.34, 0.40, and 0.44 for athlete intake of EPA, DHA, and EPA+DHA, respectively, with blood EPA, DHA, and the omega-3 index being considered [29]. Another study developed a short FFQ for assessing the intake of EPA, DHA, and EPA+DHA, resulting in CCs of 0.48, 0.73, and 0.73, respectively, with relevant erythrocyte biomarkers [30]. In this study, the CCs between the intake of EPA, DHA, and EPA+DHA and corresponding serum biomarkers were 0.389, 0.52, and 0.463, respectively, which are comparable to or slightly lower than those reported in previous studies. While the FFQ utilized in this study was not specifically tailored for evaluating omega-3 polyunsaturated fatty acid intake as in the aforementioned studies, it displayed acceptable to moderate validity for EPA and DHA, which indicated that the FFQ was dependable in recalling food items rich in omega-3 polyunsaturated fatty acids, mainly freshwater fish and seafood. However, the validity coefficient for the food group "fish and shellfish" appeared less satisfactory (0.287) against the 3DWFR, likely due to the limitations of the random 3-day weighing method in accurately capturing the average intake of these foods, as they were not consumed daily. Conversely, exogenous blood biomarkers can provide a more reliable reflection of recent nutrient intake.

In fact, when compared with 3DWFR, the CCs of food groups in this study (median CC of 0.292) were lower than those reported in previous researches [11,13], a result that could be attributed to factors such as questionnaire length, reference method, food group classification, and cultural dietary differences. Nevertheless, acceptable CCs were found for cereals and potatoes, poultry and meat, eggs, and milk and dairy products. These foods were commonly consumed by athletes, with substantial daily intake and stable consumption frequencies, reducing the likelihood of recall bias and thus resulting in higher correlations. In comparison, the correlations for vegetables and legumes and nuts were considerably lower, and energy adjustment failed to improve the CCs for these two food groups. The lower CC value observed for vegetables may stem from athletes subjectively increasing their consumption of these perceived ’healthy’ foods during the weighing period. According to our knowledge, athletes generally undervalue vegetable intake, with their daily intake frequently below recommended levels. The lower CC value for legumes and nuts may be due to their sporadic and random intake patterns, making it challenging for the 3-day weighing method to accurately capture the consumption of this food group. Future research may require more reliable reference methods, such as increased weighing frequency, to obtain precise intake data for vegetables and legumes and nuts. Among food groups, oils displayed the lowest correlations, with both their crude and energy-adjusted CCs being negative. This observation aligns with previous findings for Japanese athletes [13], likely attributed to the underestimation of oil quantities in Chinese-style frying or stir-frying by the FFQ. Thus, adjustments are warranted when evaluating oil intake using this FFQ, requiring the inclusion of items pertaining to cooking methods.

Although the correlation analysis results indicated the acceptable validity of this FFQ, it was evident that it tended to overestimate the intake of most nutrients and exhibited substantial biases in estimating food group intakes (Table 2), results that are consistent with numerous previous findings [11,29,31–33]. The bias in absolute intake estimation is an inherent limitation of FFQs, warranting careful consideration when utilizing an FFQ to evaluate nutrient or food group intakes at the individual level. However, the primary role of an FFQ is to rank the nutrient intakes of populations for the purpose of the rational classification of study subjects [8], and such classification will be crucial for subsequent investigation of the relationship between athletes’ dietary patterns and exercise performance. Therefore, it was wise to integrate consistency assessments within the framework of validity assessment. Quintile cross-classification consistency, weighted kappa coefficients, and Bland–Altman plots were employed in this study to evaluate the consistency of our FFQ against a reference method. The median percentage of nutrients and food groups classified within the same or adjacent quintiles stood at 61%, while the median misclassification rates for energy and nutrient components were 2%, and this rate for food groups was 3%. These findings are very similar to the results of FFQ validation studies conducted on Japanese athletes [11], indicating good quintile agreement with the reference method. Moreover, the weighted kappa values fell within acceptable thresholds (0.2–0.6) for the majority of nutrients and food groups. The Bland–Altman plots showed that the majority of data points for macronutrient and food groups were within the limits of agreement (LOA) near the mean intake, with fewer than 10% of the data points distributed outside the LOA, further corroborating the high degree of consistency between the FFQ and 3DWFRs.

Finally, the reproducibility of two FFQs within a one-month interval was tested. In this study, the reproducibility CCs and ICCs ranged from 0.5 to 0.8 for most nutrients and food groups, comparable to previous research conducted on athletes and other healthy adult populations [10,13,34–36]. Additionally, in terms of the absolute intakes, the second FFQ underestimated most nutrients and food groups compared to the first FFQ. This discrepancy could be attributed to the inclusion of 3DWFRs between the administration of the two FFQs, providing participants with a more immediate understanding of food portion sizes and subsequently allowing for more accurate adjustments to their questionnaire responses. This was also why we utilized the data from the first FFQ in the assessment of relative validity to more accurately reflect the results of the validity tests.

There are several limitations in our study. Firstly, the athletes included in our re-search were exclusively from a specific region in China, namely, Shanghai. Due to regional dietary variations, the results of this study may not be directly applicable to athletes from other regions. Secondly, this study was conducted during the winter training period, and caution should be exercised when extrapolating the findings to other seasons of the year, as the results may be influenced by seasonal variations in food availability. Thirdly, the reference period for the reference methods (3DWFRs and blood biomarkers) and FFQ did not overlap, possibly affecting the results for relative validity. Lastly, the number of items in the FFQ was limited and might not cover a comprehensive range of food items. Nonetheless, the brevity of the FFQ was also a strength of this study. Shortening questionnaire completion time could maximize support from athletes and coaches, thereby increasing the likelihood of obtaining an adequate sample size. Furthermore, the final validity and reproducibility results of this study were comparable to those from other similar studies, affirming the effectiveness of our short FFQ in both item selection and food list composition.

In conclusion, the present study validated the first short FFQ specifically developed for Chinese (Shanghainese) professional athletes. Including items related to protein powder, sports drinks, and specialized nutrition supplements in the short FFQ is essential for athletes, as these items contribute significantly to the intake of both macro- and micronutrients. This short FFQ demonstrated acceptable validity and reproducibility for most nutrients and food groups, with good consistency against reference methods. It can serve as a useful tool for assessing the habitual intakes of athletes in specific training periods, including macronutrients and key nutrients such as vitamin D and omega-3 polyunsaturated fatty acids. Moreover, it will serve as a pivotal tool for further exploring the intricate relationship between dietary patterns and the health and/or performance of athletes.

## Supporting information

S1 Table Characteristics of the study participants: A gender-based comparison. ^a^Athletes competing at the national level who are eligible to compete in comprehensive competitions and championships held by national sports bureaus and satisfy the criteria. ^b^The top 12 competitors who are eligible to compete in comprehensive competitions and championships organized by provincial and local sports bureaus and have been designated as first level athletes. ^c^The top 24 competitors who are eligible to compete in comprehensive competitions and championships organized by provincial and local sports bureaus and have been designated as second level athletes.
(DOCX)

S2 Table Bland Altman plot analysis for testing the agreement of 3DWFRs and FFQ1 (n = 97). LOA: Limit of agreement.
(DOCX)

S1 File Raw data of the study. (XLSX)
