# Supplementary material for: Relative validity and reproducibility of a short semi-quantitative food frequency questionnaire for Chinese athletes
Source: PLoS One. 2025 Jan 9;20(1):e0317370. doi: 10.1371/journal.pone.0317370 (PMC11717214; doi:10.1371/journal.pone.0317370)
Supplement: S1 Table — aAthletes competing at the national level who are eligible to compete in comprehensive competitions and championships held by national sports bureaus and satisfy the criteria. bThe top 12 competitors who are eligible to compete in comprehensive competitions and championships organized by provincial and local sports bureaus and have been designated as first level athletes. cThe top 24 competitors who are eligible to compete in comprehensive competitions and championships organized by provincial and local sports bureaus and have been designated as second level athletes. (DOCX) [file pone.0317370.s001.docx]

Table S1. Characteristics of the study participants: a gender-based comparison.

| Sex | Characteristics | Results |
| --- | --- | --- |
| Male (n, %) |  | 49 (48.04) |
|  | Age, y (mean±SD) | 19.90 ±3.46 |
|  | Height, cm (mean±SD) | 179.70 ±7.45 |
|  | Weight, kg (mean±SD) | 76.97 ±13.19 |
|  | Body Mass Index, kg/m^2^ (mean±SD) | 23.77 ±3.41 |
|  | Sports Event (n, %) |  |
|  | Cycling | 14 (28.57) |
|  | Hand ball | 3 (6.12) |
|  | Shooting archery | 13 (26.53) |
|  | Wushu | 8 (16.33) |
|  | Track and field | 4 (8.16) |
|  | Volleyball | 3 (6.12) |
|  | Boxing | 3 (6.12) |
|  | Swimming | 1 (2.04) |
|  | Athlete Class (n, %) |  |
|  | National^a^ | 23 (46.94) |
|  | 1^st^ level^b^ | 13 (26.53) |
|  | 2^nd^ level^c^ | 13 (26.53) |
|  | Training years, y (mean±SD) | 8.42 ±3.80 |
|  | Weekly Training Time, h (mean±SD) | 26.34 ±9.92 |
| Female (n, %) |  | 53 (51.96) |
|  | Age, y (mean±SD) | 19.13 ±3.78 |
|  | Height, cm (mean±SD) | 168.51 ±5.89 |
|  | Weight, kg (mean±SD) | 60.50 ±12.64 |
|  | Body Mass Index, kg/m^2^ (mean±SD) | 21.28 ±4.16 |
|  | Sports Event (n, %) |  |
|  | Cycling | 3 (5.66) |
|  | Hand ball | 5 (9.43) |
|  | Shooting archery | 12 (22.64) |
|  | Wushu | 5 (9.43) |
|  | Track and field | 10 (18.87) |
|  | Boxing | 2 (3.77) |
|  | Swimming | 4 (7.55) |
|  | Badmiton | 6 (11.32) |
|  | Gymnastics | 6 (11.32) |
|  | Athlete Class (n, %) |  |
|  | National^a^ | 25 (47.17) |
|  | 1^st^ level^b^ | 16 (30.19) |
|  | 2^nd^ level^c^ | 12 (22.64) |
|  | Training years, y (mean±SD) | 8.56 ±3.43 |
|  | Weekly Training Time, h (mean±SD) | 29.43 ±8.39 |

^a^ Athletes competing at the national level who are eligible to compete in comprehensive competitions and championships held by national sports bureaus and satisfy the criteria. ^b^ The top 12 competitors who are eligible to compete in comprehensive competitions and championships organized by provincial and local sports bureaus and have been designated as first level athletes. ^c^ The top 24 competitors who are eligible to compete in comprehensive competitions and championships organized by provincial and local sports bureaus and have been designated as second level athletes.
