# Supplementary material for: Relative validity and reproducibility of a short semi-quantitative food frequency questionnaire for Chinese athletes
Source: PLoS One. 2025 Jan 9;20(1):e0317370. doi: 10.1371/journal.pone.0317370 (PMC11717214; doi:10.1371/journal.pone.0317370)
Supplement: S2 Table — LOA: Limit of agreement. (DOCX) [file pone.0317370.s002.docx]

Table S2. Bland Altman plot analysis for testing the agreement of 3DWFRs and FFQ1 (n = 97).

|  | **Mean Difference** | **SD of Mean Difference** | **Lower LOA** | **Upper LOA** | **%within LOA** |
| --- | --- | --- | --- | --- | --- |
| **Energy and nutrients**  Energy | 108.80 | 1047.00 | -1943.33 | 2160.92 | 95.88 |
| Protein | 23.84 | 72.49 | -118.24 | 165.92 | 95.88 |
| Fat | -7.66 | 51.83 | -109.25 | 93.93 | 95.88 |
| Carbohydrate | 19.42 | 120.88 | -217.50 | 256.35 | 94.85 |
| Dietary fiber | 0.75 | 5.43 | -9.90 | 11.40 | 95.88 |
| Cholesterol | 64.20 | 485.98 | -888.33 | 1016.73 | 92.78 |
| Vitamin A | -73.20 | 314.56 | -689.73 | 543.33 | 92.78 |
| Vitamin B1 | 0.56 | 1.04 | -1.47 | 2.60 | 93.81 |
| Vitamin B2 | 0.36 | 0.82 | -1.25 | 1.96 | 93.81 |
| Niacin | 9.93 | 17.94 | -25.22 | 45.09 | 91.75 |
| Vitamin C | 48.23 | 82.78 | -114.02 | 210.48 | 93.81 |
| Vitamin E | -0.22 | 16.74 | -33.04 | 32.59 | 95.88 |
| Vitamin D | 95.05 | 363.59 | -617.59 | 807.70 | 96.91 |
| Calcium | 74.64 | 433.49 | -775.00 | 924.28 | 93.81 |
| Phosphorus | 260.09 | 699.07 | -1110.09 | 1630.26 | 95.88 |
| Potassium | 150.47 | 1112.69 | -2030.40 | 2331.35 | 95.88 |
| Sodium | -1137.78 | 1934.01 | -4928.45 | 2652.89 | 94.85 |
| Magnesium | 40.85 | 169.74 | -291.85 | 373.54 | 95.88 |
| Iron | -0.14 | 12.45 | -24.55 | 24.27 | 93.81 |
| Zink | 4.01 | 11.75 | -19.02 | 27.05 | 93.81 |
| Selenium | 23.76 | 50.80 | -75.81 | 123.33 | 96.91 |
| Copper | 0.76 | 1.53 | -2.24 | 3.75 | 94.85 |
| Manganese | 0.79 | 2.31 | -3.74 | 5.31 | 94.85 |
| **Food groups**  Cereals and potatoes | -73.63 | 241.94 | -547.84 | 400.58 | 95.88 |
| Vegetables | -57.59 | 155.12 | -361.63 | 246.45 | 93.81 |
| Fruits | 169.38 | 217.68 | -257.28 | 596.04 | 92.78 |
| Poultry and meat | 125.39 | 271.95 | -407.62 | 658.41 | 92.78 |
| Fish and shellfish | -5.92 | 154.35 | -308.45 | 296.61 | 93.81 |
| Eggs | -50.04 | 74.96 | -196.97 | 96.89 | 94.85 |
| Milk and dairy products | 38.76 | 200.39 | -354.01 | 431.53 | 93.81 |
| Legumes and nuts | 14.77 | 53.05 | -89.21 | 118.75 | 96.91 |
| Oils | -6.98 | 24.67 | -55.34 | 41.38 | 96.91 |
